# In-Vitro Antioxidant Properties of Lipophilic Antioxidant Compounds from 3 Brown Seaweed

**DOI:** 10.3390/antiox8120596

**Published:** 2019-11-28

**Authors:** Gaurav Rajauria

**Affiliations:** School of Agriculture and Food Science, University College Dublin, Lyons Research Farm, Celbridge, Co. Kildare W23 ENY2, Ireland; gaurav.rajauria@ucd.ie; Tel.: +353-1-601-2167

**Keywords:** lipophilic antioxidant, solvent blending, macroalgae, LC-ESI-MS/MS, carotenoid pigment, anthocyanin, chlorophyll derivative

## Abstract

Lipophilic compounds of seaweed have been linked to their potential bioactivity. Low polarity solvents such as chloroform, diethyl ether, *n*-hexane and their various combinations were used to extract the lipophilic antioxidants from brown seaweed namely *Himanthalia elongata, Laminaria saccharina* and *Laminaria digitata*. An equal-volume mixture of chloroform, diethyl ether and *n*-hexane (Mix 4) gave the highest total phenol (52.7 ± 1.93 to 180.2 ± 1.84 mg gallic acid equivalents/g), flavonoid (31.9 ± 2.65 to 131.3 ± 4.51 mg quercetin equivalents/g), carotenoid (2.19 ± 1.37 to 3.15 ± 0.91 μg/g) and chlorophyll content (2.88 ± 1.08 to 3.86 ± 1.22 μg/g) in the tested seaweeds. The extracts were screened for their potential antioxidant capacity and the extracts obtained from the selected solvents system exhibited the highest radical scavenging capacity against 2,2′-diphenly-1-picrylhydrazyl radical (EC_50_ 98.3 ± 2.78 to 298.8 ± 5.81 mg/L) and metal ions (EC_50_ 228.6 ± 3.51 to 532.4 ± 6.03 mg/L). Similarly, the same extract showed the highest ferric reducing antioxidant power (8.3 ± 0.23 to 26.3 ± 0.30 mg trolox equivalents/g) in all the seaweeds. Rapid characterization of the active extracts by liquid chromatography coupled with photodiode array detector and electrospray ionization tandem mass spectrometry (LC-PDA–ESI-MS/MS) identified cyanidin-3-*O*-glucoside, fucoxanthin, violaxanthin, *β*-carotene, chlorophyll *a* derivatives and chlorophyll *b* derivatives in the tested seaweed. The study demonstrated the use of tested brown seaweed as potential species to be considered for future applications in medicine, cosmetics and as nutritional food supplement.

## 1. Introduction

The concepts of nutrition are changing rapidly as consumers all over the world have become more cautious regarding nutritionally healthier food and its ingredients. Recently, a great interest in using natural plant-derived bioactive compounds in foods, cosmetics and pharmaceuticals has arisen, due to their nutritional and therapeutic effects [1,2]. The epidemiological and observational literatures suggest that free radicals play an important role in affecting human health by causing cancers or age associated neurodegenerative diseases. However, antioxidant-rich foods have shown their relevance in the prevention of these diseases by mitigating the harmful free radicals or reactive oxygen species (ROS) [3]. Chemical compounds such as butylated hydroxytoluene (BHT; E-321), butylhydroxyanisole (BHA; E-320) and ascorbic acid (E-300) are commonly used as synthetic antioxidants in food products to improve the product quality and shelf life. However, due to possible toxicity of synthetic antioxidants as well as consumer preference towards natural substances, natural antioxidants are considered safe and more acceptable for use as ingredients in functional foods, nutraceuticals and cosmetics [4,5]. Among the most studied classes of natural antioxidants, phenolic compounds and carotenoid pigments are widely distributed in the plant kingdom and have received much attention for their high antioxidant activity [6]. Although these functional ingredients are not restricted to terrestrial resources, plants in general and seaweeds (marine plants) in particular, are good sources of natural antioxidants. Seaweed grows in extreme environmental conditions thus producing a variety of antioxidant compounds to counteract environmental stresses [7]. The most important naturally occurring seaweed substances showing antioxidant properties are polyphenols, phlorotannins, flavonoids, carotenoids, fatty acids, polysaccharides and amino acids, which in varying proportion and quantities, are reported in different seaweed species [8,9,10]. A variety of in vitro studies have shown that lipophilic compounds such as carotenoid pigments and some polyphenols and flavonoids exhibit strong antioxidant activity [11,12,13,14]. These compounds are capable of acting as primary antioxidants by reacting with free radical species or could act as secondary antioxidants (metal chelator) by blocking the generation of hypervalent metal forms [15]. Such antioxidant activities of carotenoids and polyphenols may protect cells from ROS-induced cellular damage, thereby reducing the risk of diseases associated with oxidative stress [16].

Multiple compounds from hundreds of algal species have been studied up until now and a range of compounds possessing antioxidant properties have been discovered. Among these compounds, some compounds are of polar or hydrophilic nature (e.g., phlorotannins), some are semi-polar (e.g., phenolic acids and simple flavonoids), some and others are non-polar or lipophilic in nature (e.g., carotenoids, fatty acids). They may also exist as complexes with sugar, proteins and other cell membrane components; which make them quite insoluble and a selective solvent system is required to solubilize and extract them [17]. Extractability of bioactive compounds is associated with the polarity of solvents (polar/semi-non-polar) used, as well as their complexity with other constituents. Finding a solvent system suitable for the extraction of all classes or a specific class of antioxidant is restricted by the chemical nature of these bioactive compounds. These bioactives are present in matrices as a complex mixture of compounds that provide a cocktail of many active components present in the free, esterified, glycosylated and bound states as conjugates with other components that lead to the formation of insoluble complexes. The solubility of these compounds is administered by the nature of raw material, degree of polymerization and the polarity of solvent used [1]. Therefore, the extraction of these active ingredients from seaweed matrices is the key step to utilizing them for pharmaceutical, cosmeceutical, and foods as well as nutraceutical preparations. Thus, to obtain extracts enriched in lipophilic compounds, it is of critical importance to select efficient extraction solvent systems to improve their extractability and to maintain stability. Additionally, extraction solvent can have a significant effect on the performance of antioxidant reaction mechanisms which can change the chemical behavior of antioxidant compounds [18]. Therefore, there is no uniform or completely satisfactory procedure that is suitable for extraction of all compounds or a specific class of compounds from plant materials [17]. Thus, the objective of the present study was to select the appropriate solvent system that is capable of extracting lipophilic compounds from Irish brown seaweeds and to evaluate the antioxidant capacity and phytochemical constituents of those extracts. Seaweeds were extracted with semi/non-polar solvents and their mixtures, in order to get the lipophilic antioxidant compounds. The crude extracts were screened for total polyphenol, flavonoid, chlorophyll and carotenoid content along with antioxidant reducing power and potential radical scavenging capacity against 2,2′-diphenyl-1-picrylhydrazyl (DPPH) radicals and metal-ions. The identification and characterization of antioxidant compounds were carried out by using liquid chromatography coupled with electrospray ionization tandem mass spectrometry (LC-ESI-MS/MS) and UV-visible spectroscopy.

## 2. Materials and Methods

### 2.1. Chemicals, Solvents and Standards

Folin-Ciocalteu’s phenol reagent, 2,2′-diphenyl-1-picrylhydrazyl (DPPH), 3-(2-pyridyl)-5,6-diphenyl-1,2,4-triazine-4′,4′′-disulphonic acid monosodium salt (ferrozine), 2,4,6-tripyridyl-s-triazine (TPTZ) and 6-hydroxy-2,5,7,8-tetramethylchroman-2-carboxylic acid (Trolox) were purchased from Sigma-Aldrich Chemical Co. (Steinheim, Germany). For LC-MS analysis, solvents such as water, methanol and acetonitrile were chromatography grade which was purchased from Fisher Chemicals (Thermo Fisher Scientific Inc., Dublin, Ireland). Authentic standards including L-ascorbic acid, gallic acid, quercetin, cyanidin 3-glucoside, fucoxanthin and violaxanthin were purchased from Sigma-Aldrich Chemical Co. (Arklow, Co. Wicklow, Ireland). All other chemicals used in the study were analytical grade and purchased from Sigma-Aldrich Chemical Co. (Ireland).

### 2.2. Seaweed Materials and Extraction Procedure

**Figure 1:**
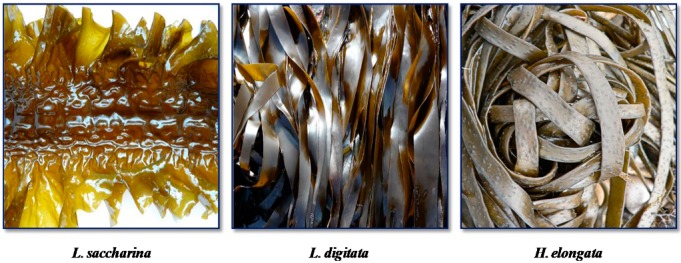
Images of brown Irish seaweeds studied.

Irish brown seaweeds (Figure 1) used in this study namely, *Laminaria digitata*, *Laminaria saccharina* and *Himanthalia elongata* (*Phaeophyta*) were purchased from Quality Sea Veg., Co Donegal, Ireland. Among the tested seaweeds, *L. digitata* and *L. saccharina* (also known as *Saccharina latissima*) are large conspicuous dark brown and yellow brown kelp, commonly found down to a maximum depth of 20 m and 30 m in clear waters respectively. The stipes of *L. digitata* are smooth, flexible and oval in cross section while *L. saccharina* has a long undivided frond with a distinct bullations and a distinctive frilly undulating margin. Both are usually found attached to bedrock or other suitable hard substrata in the low water in Intertidal pools and occasionally in the shallow subtidal zones. *H. elongata* is a light yellow-brown fucoid species which has long, narrow, strap-like branched fronds with basal mushroom-like buttons. It is found attached to rocks or hard substrata on moderately semi-wave-exposed shore. Seaweed species were harvested and collected in winter (January/February), washed thoroughly with fresh water to remove epiphytes, eliminate salt, sand or shells and stored at −20 °C until analysis.

Extraction of lipophilic components from seaweed was carried out by crushing the fresh sample with liquid nitrogen followed by extraction with semi/non-polar organic solvents such as chloroform, diethyl ether, *n*-hexane and thereof mixtures according to the method described earlier [19,20]. The mixtures of solvents used were; Mix 1 (*n*-hexane and diethyl ether), Mix 2 (*n*-hexane and chloroform), Mix 3 (diethyl ether and chloroform) and Mix 4 (*n*-hexane, diethyl ether and chloroform). All the solvents were mixed either 1:1 (*v*/*v*) or 1:1:1 ratio (*v*/*v*/*v*) depending upon the mixtures, and dielectric constant (*ε*) of individual solvent as well as their mixture were taken into account. The extracted samples were filtered with Whatman #1 filter paper and centrifuged at 9168× *g* (Sigma 2–16PK, SartoriusAG, Gottingen, Germany) for 15 min. The resulting supernatant was evaporated to dryness, and the dried lipophilic extract was dissolved in HPLC (high performance liquid chromatography) grade methanol for further analysis. The whole extraction procedure was carried out under dark conditions to minimize the possibility of oxidation/degradation of antioxidant compounds by light.

### 2.3. Phytochemical Constituent Analysis

Crude lipophilic extracts of seaweed were screened for total phenolic content (TPC), total flavonoid content (TFC), total chlorophyll content (TChC) and total carotenoid content (TCC). TPC was determined according to Ganesan et al. [9]. Samples were read at 720 nm and the results were expressed as mg gallic acid equivalents (GAE)/g dry weight (dw) extract. TFC and TTC were determined according to Liu et al. [21]. Samples were read at 510 nm and 500 nm, and the results were expressed as mg quercetin equivalents (QE)/g extract (dw) and mg (+)-catechin equivalents (ChE)/g extract (dw), respectively. TChC and TCC were determined according to Arnon (1949) and Kirk and Allen (1965) respectively. For chlorophyll, the samples were read at 645 nm and 663 nm, and total content was calculated by using Equation (1), while for total carotenoids, the absorbance of the same chlorophyll samples was recorded at 480 nm and content was calculated by using Equation (2).

TChC (µg/g; dw) = 20.2 × (*A*_645_) + 8.02 × (*A*_663_) (1)

where *A* = Absorbance at respective wavelength

TCC (μg/g; dw) = *A*_480_ + (0.114 × *A*_663_) − (0.638 × *A*_645_) (2)

where, *A* = Absorbance at respective wavelength

### 2.4. Antioxidant Capacity Analysis

#### 2.4.1. DPPH Radical Scavenging Capacity Assay

This assay was carried out according to the method reported earlier [22]. Ascorbic acid was used as a standard and the absorbance of the standard or samples was recorded at 517 nm using a 96-well plate reader. The ability of samples to scavenge the DPPH radical was calculated using Equation (3):

DPPH radical scavenging capacity (%) = [1 − (*A*_sample_ − *A*_sample blank_)/*A*_control_] × 100 (3)

where, *A* = Absorbance of sample/sample blank or control

#### 2.4.2. Ferric Reducing Antioxidant Power (FRAP) Assay

Total antioxidant reducing power of various extracts of seaweed was measured using modified FRAP assay [23]. Trolox was used as a standard and the absorbance of the standard or samples was recorded at 593 nm, and the results were expressed as mg trolox equivalents (TE)/g extract (dw).

#### 2.4.3. Metal Ion-Chelating Ability Assay

The chelating ability of metal ion (ferrous ion) by seaweed extracts was estimated using the original method of Decker and Welch [24] with minor modifications. This assay is based upon the formation of blue colored ferrous ion-ferrozine complex which has a maximum absorbance at 562 nm. EDTA (ethylenediaminetetraacetic acid) was used as a standard compound. The percentage of inhibition of ferrozine-Fe^2+^ complex formation was calculated using Equation (3).

### 2.5. Characterization of Lipophilic Compounds using Liquid Chromatography Mass Spectrometry (LC–MS)

Antioxidant compounds in the lipophilic extracts were analyzed on 6410 Triple Quadrupole LC/MS, fitted with Agilent 1200 series LC, G1315B variable-wavelength photodiode array (PDA) detector and MassHunter Workstation software (version B.04.00, Agilent Technologies, Santa Clara, CA, USA). The separation was performed at 25 °C using an Atlantis C-18 (250 × 4.6 mm, 5 µm particle size) column fitted with a suitable C-18 (4.0 × 3.0 mm) guard cartridge (Waters, Dublin, Ireland). The mobile phase consisting of ternary solvents of acetonitrile/methanol/water (75:15:10, *v*/*v*/*v*) containing 1.0 g/L ammonium acetate, eluted at 1.0 mL/min for 25 min, was adopted from Sugawara [25]. The injection volume of 10 µL was kept constant for samples and standard compounds. UV-vis absorption of the selected extracts was recorded from 190 to 600 nm using LC-PDA detector and the *λ*_max_ (absorption maxima) of each peak was noted. Peaks assignments were made by comparing the UV/visible spectra of analytes to standard compounds, and available literature. Mass spectral data were recorded on positive ionization mode using electrospray ionization (ESI) interface with 3.5 kV capillary voltage, 120 V fragmentor voltage and 10 eV collision energy in the mass range of *m*/*z* 100–1000. Nitrogen gas was used as the nebulizer and drying gas with 50 psi pressure, 10 L/min flow rate, 350 °C drying temperature and 35 nA capillary current. The identification of the peaks was carried out using mass spectral data of standard compounds where possible. Identification of remaining peaks was based on UV-visible spectral (*λ*_max_) characteristics and the results were compared with the literature when no standards were available.

### 2.6. Statistical Analysis

Statistical analyses were carried out using STATGRAPHICS Centurion XV software (version XV, Statgraphics Technologies, Inc., The Plains, VA, USA). All the experiments were carried out in triplicate and repeated twice. Results are expressed as mean ± standard deviation. Statistical differences between antioxidant activities or phytochemical content of extracts were determined using Analysis of Variance (ANOVA) followed by Least Significant Difference (LSD) testing. Differences were considered statistically significant when *p* < 0.05.

## 3. Results and Discussion

### 3.1. Phytochemical Content in Lipophilic Extracts

It is widely accepted that bioactive compounds can be classified by their solubility into hydrophilic and lipophilic compounds. Similar to hydrophilic compounds, lipophilic compounds also play an important role in a wide spectrum of biochemical and physiological processes [11]. These lipophilic compounds can be extracted with semi/non-polar solvents in plants wherein polarity of the solvents play a significant role in the resulting yield, extractability and biological activity of bioactive compounds. In this study, various organic solvents and their combinations with varying dielectric constant were used to extract lipophilic compounds from 3 brown seaweed.

Results from Table 1 have shown a considerable variation in the extraction yield among the extracts recovered from various low polarity solvents and their mixtures. The extraction yield varied from 0.05% to 0.20% among all the tested seaweeds. The extracts recovered from *n*-hexane and Mix 1 solvents exhibited significantly (*p* > 0.05) the highest and the lowest extraction yield respectively. It is reported that low polarity (semi/non-polar) solvents generally give less extraction yield as compared to polar solvents [26] which is in agreement with the results obtained in this study.

Phytochemical content was majorly affected by the polarity of the extraction solvents as depicted in Table 1. In each of the tested seaweed, TPC from all the extracts was significantly different (*p* > 0.05) among the tested solvent systems. The extracts obtained from *n*-hexane exhibited the lowest TPC (varied from 7.7 ± 0.64 to 14.1 ± 0.79 mg GAE/g) while the extracts recovered from Mix 4 (*n*-hexane, diethyl ether and chloroform) solvents showed the highest TPC (ranging from 52.7 ± 1.93 to 180.2 ± 1.84 mg GAE/g), in all the species studied. The highest and significantly different (*p* < 0.05) amount of TPC was obtained in *H. elongata* followed by *L. saccharina* and *L. digitata* with the Mix 4 solvent system.

In the case of total flavonoid, the results showed that TFC in seaweeds varied considerably with the solvent polarity. The TFC of extracts obtained from different low polarity solvents and their mixtures ranged from 11.3 ± 2.5 to 131.3 ± 4.51 mg QE/g in *H. elongata*, 6.9 ± 0.88 to 56.3 ± 1.77 mg QE/g in *L. saccharina* and 4.4 ± 0.88 to 31.9 ± 2.65 mg QE/g in *L. digitata*. The extract from Mix 4 solvents exhibited the highest and significantly different (*p* < 0.05) TFC in *H. elongata* followed by *L. saccharina* and *L. digitata*. However, the extract obtained from *n*-hexane showed the lowest TFC in all the seaweed species (Table 1). There was no significant difference observed in TFC between the extract of Mix 1 (*n*-hexane: diethyl ether) and Mix 2 (*n*-hexane: chloroform) solvents within an individual seaweed species.

Pigments such as carotenoids play an important role in seaweed reproduction and are responsible for different colors. Fucoxanthin, a major pigment of brown seaweeds, is one of the most abundant carotenoids in nature and constitute 10% to total carotenoid production [27]. It is an orange-colored pigment, found along with chlorophyll pigment (*a* and *c*) and *β*-carotene, to give a brown or olive-green color to brown seaweed [28,29,30]. Numerous studies have shown that brown seaweed pigments such as fucoxanthin, violaxanthin and *β*-carotene have substantial applications in human health. These pigments have been explored for its potential bioactivities including antioxidant, anti-inflammatory, anticancer, anti-obese and antidiabetic property [14,31,32]. Table 1 shows that the spectrophotometric measurement of total carotenoids and total chlorophyll content in various extracts of 3 brown seaweed studied. The results revealed that the Mix 4 solvent system produced significantly higher TCC in *H. elongata* followed by *L. digitata* and *L. saccharina* whereas *n*-hexane extracts presented the lowest values among the tested seaweed. In the case of chlorophyll content, extracts from chloroform (instead of Mix 4 solvents) exhibited the highest TChC while extracts recovered from Mix 2 solvents showed the lowest TChC (*p* < 0.05), among the tested seaweeds and their extracts (Table 1). It was observed that the chloroform extract of *L. digitata* exhibited the highest TChC (*p* < 0.05) whereas *H. elongata* extract presented the lowest TChC.

Furthermore, upon analyzing TPC, TFC, TChC and TCC results against the polarity or dielectric constant of extraction solvents and their mixtures, an interesting relationship was observed. The results interpreted that the phytochemical content was primarily affected by the semi/non-polar extraction solvents. The dielectric constant of solvents and their mixtures was in the range of 5.0 to 2.0 with the following decreasing order: chloroform (5.0) > Mix 2 (4.7) > diethyl ether (4.3) > Mix 4 (3.8) > Mix 3 (3.5) > Mix 1 (3.2) > *n*-hexane (2.0). The dielectric constant of the mixed solvents is calculated on the basis of percentage (*v*/*v*) of each solvent used for the combinations. The dielectric constant of a solvent is an index of its polarity, and an increase in polarity shows a similar increase in the dielectric constant [10]. Mixing of solvents with different polarities is an approach to form a solvent system of optimum polarity to extract the various bioactive compounds. This approach is referred to as “solvent blending” or “co-solvency” and uses the dielectric constant as a guide to develop the co-solvent system [33]. The results indicated that the polarity/dielectric constant of Mix 4 solvent system (*n*-hexane, diethyl ether and chloroform) was more selective to the lipophilic phenolic compounds present in selected seaweeds than the other tested solvents and their mixtures. These findings are also in agreement with the report of Sahreen [34] wherein a range of polarity solvents gave different values of TPC, TFC and extraction yield. Furthermore, these findings also suggest that yield may not be a good indicator of phytochemical content of extracts based on the fact that phytochemical content was the lowest in the *n*-hexane extract, but had the highest extraction yield in all the studied seaweeds, which agrees with the previous reported results [35].

This study, as well as other previously reported publications [1,17,36], clearly illustrates that it is essential to systematically evaluate and optimize the extraction solvent composition for accurate and reproducible estimation of structurally diverse antioxidant compounds from different plants. In the present study, the highest recoveries of lipophilic antioxidants from seaweeds samples were obtained from Mix 4 solvents mixture using a solvent extraction technique.

### 3.2. Antioxidant Capacity of Lipophilic Extracts

**Figure 2:**
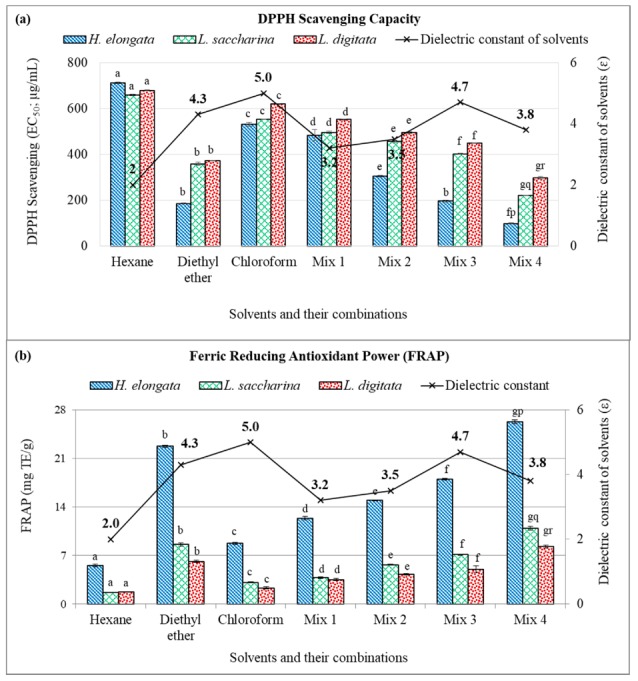
2,2′-diphenyl-1-picrylhydrazyl (DPPH) radical scavenging capacity (**a**) and ferric reducing antioxidant power (**b**) of the Irish brown seaweeds extracts obtained from semi/non-polar organic solvents and thereof mixtures (1:1 or 1:1:1, *v*/*v*/*v*). [Mix 1: *n*-hexane and diethyl ether; Mix 2: *n*-hexane and chloroform; Mix 3: diethyl ether and chloroform; Mix 4: *n*-hexane, diethyl ether and chloroform]. [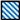: *H. elongata*; 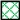: *L. saccharina*; 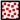: *L. digitata*]. Data are expressed as mean ± SD (n = 6). Ferric Reducing Antioxidant Power (FRAP) values are expressed as mg Trolox equivalent (TE)/g extract (dry weight). Letters (a–g) on each bar are significantly different (*p* < 0.05) for various solvents, for each individual species. Letters (p–r) on bars at a specific solvent (Mix 4) are significantly different (*p* < 0.05) among the three species. Mix 1: *n*-hexane and diethyl ether; Mix 2: *n*-hexane and chloroform; Mix 3: diethyl ether and chloroform; Mix 4; *n*-hexane, diethyl ether and chloroform. All the solvents were mixed either 1:1 or 1:1:1 ratio (*v*/*v*).

**Figure 3:**
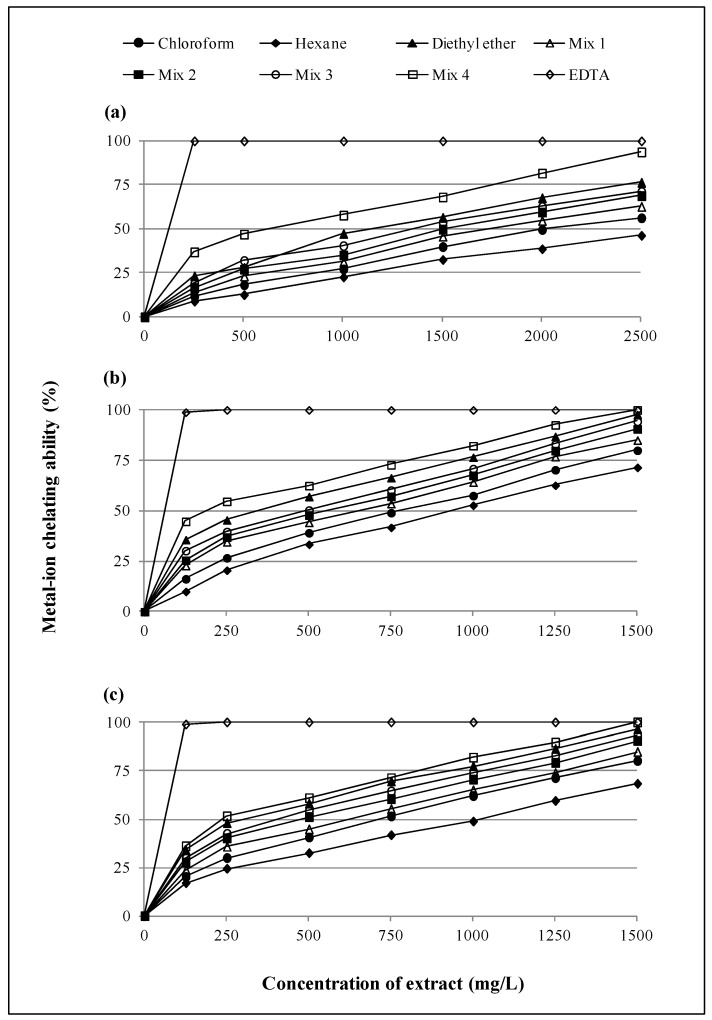
Metal-ion chelating ability of ethylenediaminetetraacetic acid (EDTA) standard and the Irish brown seaweeds extracts obtained from semi/non-polar solvents and mixtures (1:1 or 1:1:1, *v*/*v*/*v*) thereof (**a**) *H. elongata*; (**b**) *L. saccharina*; (**c**) *L. digitata*. [Mix 1: *n*-hexane and diethyl ether; Mix 2: *n*-hexane and chloroform; Mix 3: diethyl ether and chloroform; Mix 4: *n*-hexane, diethyl ether and chloroform].

The lipophilic extracts of all the three tested seaweed, obtained from various solvents and their mixtures, were screened for their potential antioxidant capacity using the stable DPPH radicals, FRAP reagent and by metal ion-chelating ability assay. The results of antioxidant capacity are illustrated in Figure 2 and Figure 3. It was observed that all the seaweed exhibited a treatment effect and the scavenging of DPPH radicals by the seaweed extracts was dose-dependent. Results interpreted that EC_50_ values of all the extracts obtained from different solvents were significantly different (*p* < 0.05) in each seaweed species. The extracts from Mix 4 solvent exhibited the highest scavenging (lowest EC_50_ values) while the extracts from *n*-hexane depicted the lowest scavenging capacity (highest EC_50_ values) against DPPH radicals (Figure 2a). Among the tested seaweed, *H. elongata* showed the highest scavenging capacity (EC_50_ 98.3 ± 2.78 µg/mL) followed by *L. saccharina* (EC_50_ 222.4 ± 0.84 µg/mL) and *L. digitata* (EC_50_ 298.8 ± 5.81 µg/mL). The scavenging capacity of the standard ascorbic acid (EC_50_ 50.6 ± 0.79 µg/mL) was recorded higher than the seaweed extracts.

Reducing power appears to be related to the degree of hydroxylation and the extent of conjugation in polyphenols. The ferric reducing antioxidant power in the various extracts of brown seaweeds was studied and the results are presented in Figure 2b. The reducing ability of all the extracts were significantly different within each species and ranged from 5.5 ± 0.20 to 26.3 ± 0.30 mg TE/g dw extract in *H. elongata*, 1.6 ± 0.06 to 10.9 ± 0.29 mg TE/g dw extract in *L. saccharina* and 1.7 ± 0.06 to 8.3 ± 0.23 mg TE/g dw extract in *L. digitata*. Of the tested extracts, Mix 4 solvent extracts (*n*-hexane, diethyl ether and chloroform) exhibited the highest and statistically different (*p* < 0.05) FRAP value in *H. elongata* followed by *L. saccharina* and *L. digitata*, while the extract obtained from the *n*-hexane showed the lowest reducing power in the tested seaweed. Jiménez-Escrig [37] reported that *Fucoid* species contained more reducing power than *Laminaria* species which is in agreement with the present results.

Ferrous ions are the most powerful pro-oxidants among various species of transition metals present in food systems. Dietary antioxidants (nutrients) having the metal chelating ability, form σ-bonds with metal ions and reduce the redox potential thereby stabilizing the oxidized form of the metal ions [38]. As seen in Figure 3, the formation of Fe^2+^-ferrozine complex is disrupted in the presence of various extracts from brown seaweeds. The absorption of this complex decreased linearly in a dose-dependent manner. All the extracts had a high level of metal ion chelating ability but were significantly lower as compared to the EDTA. Among all the tested solvents, the extract from Mix 4 solvents showed the highest chelating ability (*p* < 0.05) while extracts from *n*-hexane showed the lowest metal chelating ability at any tested concentration. In contrast to FRAP and DPPH scavenging activity, the metal chelating ability was recorded higher in *Laminaria* species compared to *H. elongata.* The percentage of the metal chelating ability of all the extracts at 1000 µg/mL concentration was found to be 22.7 to 57.8% in *H. elongata*, 48.9 to 81.9% in *L. digitata* and 52.8 to 82.3% in *L. saccharina,* while standard EDTA exhibited almost 100% chelating ability even at very low (125 µg/mL) concentration (Figure 3).

Results also concluded that dielectric constant of extraction solvent has a significant role in antioxidant properties of extracted compounds. In this study, the results interpreted that the *n*-hexane (*ε* = 2.0) extracts exhibited the lowest while Mix 4 (*ε* = 3.8) extracts demonstrated the highest DPPH scavenging capacity, reducing power and metal ion chelating ability among the tested seaweed. The pattern of DPPH radical scavenging capacity shown by different solvent extracts were in the order of Mix 4 > diethyl ether > Mix 3 > Mix 1 > Mix 2 > chloroform > *n*-hexane (Figure 2a). While the arrangement of reducing power (FRAP) and metal chelating ability shown by different solvent extracts was as follows: Mix 4 > diethyl ether > Mix 3 > Mix 2 > Mix 1 > chloroform > *n*-hexane (Figure 2b and Figure 3). The recovery of lowest antioxidant activity by *n*-hexane extracts is in agreement with the previous findings wherein, *Carissa opaca* fruit extract obtained from *n*-hexane showed the lowest antioxidant capacity as compared to other higher polarity solvents indicating that solvents polarity significantly affects the antioxidant capacity [34].

### 3.3. Characterization of Lipophilic Antioxidant Compounds by LC-ESI-MS/MS

The most active extract recovered from Mix 4 solvent system was used for the identification of lipophilic antioxidant compounds from all the seaweed studied. The selected extracts were characterized by liquid chromatography coupled with mass spectrometry using positive electrospray ionization mode (LC-ESI-MS). The identification of bioactive compounds in the extracts was carried out by comparing retention time, characteristic UV/visible (UV/vis) spectra and ESI-MS fragmentation data of each separated peak with that of the authentic standard. The UV/vis spectra provide characteristic chromophore information in pigments which cannot be obtained from MS data [39]. Therefore, chlorophyll and carotenoid pigments which could not be differentiated by only MS, were characterized by a combination of UV/vis spectral data with ESI-MS.

The selected extracts exhibited good separation by reverse phase (RP) HPLC and showed 12 distinct peaks in *H. elongata*, 13 peaks in *L. saccharina* and 12 peaks in *L. digitata.* The UV-vis absorption maxima (*λ*_max_) recorded by online HPLC-PDA analyses of each peak are shown in Table 2. The absorption maxima (*λ*_max_) of peaks recorded at 280 and 532 nm corresponds to anthocyanin pigments (flavonoid derivatives) in all the extracts [40]. Compounds with typical absorptions between 400 and 500 nm with *λ*_max_ at around 425 nm corresponding to carotenoids. Absorption bands between 400–500 nm and 500–600 nm with *λ*_max_ at 430 nm and 660 nm (chlorophyll *a* derivatives) and at 450 nm and 640 nm (chlorophyll *b* derivatives) representing chlorophylls [39,41]. The characteristic UV spectra revealed the presence of 8 carotenoid derivatives, 2 chlorophyll derivatives and 1 anthocyanin pigment while 1 peak was unidentified in *H. elongata*. Similarly, *L. saccharina* extract showed the presence of 9 carotenoid derivatives, 2 chlorophyll derivatives while 2 peaks were unidentified. UV spectral data from *L. digitata* extract exhibited the occurrence of 7 carotenoid derivatives, 2 chlorophyll derivatives and 1 anthocyanin pigment while 2 peaks were unidentified. The pattern of the absorption spectrum, as well as corresponding *λ*_max_, was similar for numerous compounds extracted among 3 seaweed species studied which indicates that the tested seaweed may have a few similar compound compositions. Due to the presence of the long chromophore of conjugated double bonds, carotenoid pigments can absorb UV and visible light and provide precious information about their structure [42]. Hence, characteristic UV-visible maxima (*λ*_max_) of each individual peak of HPLC-PDA profile of selected extracts were recorded. On the basis of UV-visible spectra, these pigments can be summarized under three categories i.e., tetrapyrroles (chlorophyll derivatives), carotenoids (carotene and xanthophyll derivatives) and flavonoids (anthocyanin derivatives). Generally, both chlorophylls and carotenoids show absorption maxima within the region of 400–500 nm but only chlorophyll derivatives show an additional band within the region of 550–700 nm, which differentiate them from carotenoid derivatives [43].

Isolated lipophilic compounds from each extract were submitted for LC-ESI-MS/MS analysis. HPLC coupled to mass spectrometry with ESI proved extremely useful for peak assignment and gives a great deal of structural information and characterization of individual substances. Table 2 shows the typical ions resulting from mass spectra of lipophilic compounds obtained by LC-ESI-MS and MS/MS fragmentation. The ESI-MS spectra produced 5 protonated ([M + H]^+^) molecules at *m*/*z* 449 (peak 2), 536.9 (peak 4), 891.2 (peak 6), 601.5 (peak 7) and 659.6 (peak 11) in *H. elongata*; 3 protonated molecules at *m*/*z* 891.2 (peak 5), 601.5 (peak 7) and 659.6 (peak 12) in *L. saccharina* and 4 protonated molecules at *m*/*z* 449 (peak 1), 601.5 (peak 3), 891.2 (peak 6) and 905.5 (peak 7) in *L. digitata* (Table 2). However, MS spectra did not show any other protonated molecules from the remaining peaks of tested seaweed extracts.

Furthermore, all protonated ions were submitted for MS/MS fragmentation and their major fragmented ions are presented in Table 2. Results indicated that MS-MS fragmentation of peak 2 (*t*_R_ 2.5 min) in *H. elongata* and peak 1 (*t*_R_ 1.8 min) in *L. digitata* produced a major fragmented ion at *m*/*z* 287 [M + H − 162]^+^ due to loss of a glucose molecule from the base peak ion *m*/*z* 449 (Table 2), suggesting the presence of cyanidin-3-*O*-glucoside, which corresponds to aglycone cyaniding [44]. Anthocyanin derivatives exhibit the characteristic UV-visible maxima at a range of 515–550 nm (band I) and 275–285 nm (band II) whereas, these compounds do not show any absorption at around 400 nm [23]. Characteristic UV spectra recorded for peak 2 (*H. elongata*) and peak 1 (*L. digitata*) which showed a *λ*_max_ at 282 nm and 532 nm are in agreement with reported literature [23].

*β*-carotene, a carotenoid pigment, was identified only in *H. elongata* extract. A characteristic UV spectrum of peak 4 (*t*_R_ 3.8 min) showed a *λ*_max_ at 453 nm and 480 nm while MS data exhibited a molecular ion *m*/*z* 536.9 and upon MS/MS fragmentation, the major fragments were produced at *m*/*z* 444.2 and 430.3 corresponding to the elimination of toluene (92 u, atomic mass unit) and xylene (106 u), part of the central acyclic chain of the *β*-carotene skeleton, respectively (Table 2). This fragmentation pattern indicates the presence of extensive conjugation within the molecule or the cyclization of fragments of the polyene chain of the *β*-carotene skeleton [45]. The *β*-carotene is identified in accordance with the published results [41,46,47].

Peak 5 (*t*_R_ 5.3 min) in *H. elongata,* Peak 7 (*t*_R_ 5.0 min) in *L. saccharina* and Peak 3 (*t*_R_ 5.6 min) in *L. digitata* extracts were identified as violaxanthin according to the *λ*_max_ and its molecular ions. From MS analysis of these peaks, protonated molecular ion [M + H]^+^ was detected at *m*/*z* 601.5 and fragment ions at *m*/*z* 583.5 and 565.5 corresponding to the loss of one (18 u) and two water molecules (36 u) from the protonated ion respectively (Table 2). These assignments are consistent with the ESI-MS/MS fragmentation pattern of violaxanthin standard and are also in agreement with the mass fragmentation data described by Rivera et al. [45] wherein similar MS/MS fragmented ions (*m*/*z* 583 and 565) were recorded for violaxanthin pigment.

Peaks 11 in *H. elongata* and Peak 12 in *L. saccharina* extracts showed the same absorption spectra with the *λ*_max_ at 266 nm, 332 nm and 448 nm, but have the different retention times (*t*_R_ 15.4 and 12.6 min respectively). The MS data showed a molecular ion *m*/*z* 659.6, suggesting the presence of fucoxanthin pigment, which was confirmed by the fragment ions at *m*/*z* 641.6 and 581.5 due to the loss of water (18 u) and acetic acid along with water (78 u) from the base precursor ion respectively (Table 2). A similar ESI-MS/MS fragmentation pattern was recorded with fucoxanthin standard which confirmed the presence of fucoxanthin pigment in both seaweed extracts [14].

Identification of chlorophyll in all 3 tested extracts was confirmed by characteristic *λ*_max_, MS and MS/MS fragmentation data. Peak 6 (*t*_R_ 6.2 min) in *H. elongata,* peak 5 (*t*_R_ 3.8 min) in *L. saccharina* and peak 7 (*t*_R_ 9.4 min) in *L. digitata* extracts exhibited the same absorption spectra with the *λ*_max_ at 430 nm, 620 nm and 662 nm which corresponds to chlorophyll *a* derivatives. Different retention time of chlorophyll *a* derivative in different tested seaweeds are probably due to the presence of different epimers of chlorophyll *a* molecule. Chlorophyll epimers exhibit identical absorption spectra to the chlorophyll molecule but show different chromatographic abilities [48]. For instance, chlorophyll *a*’, an epimer of chlorophyll *a*, is less polar and appears on the longer retention time than chlorophyll *a*, because the –CHOOCH_3_ group at the C-13^2^ position in the chlorophyll a molecule is on a different plane of the C-17^3^ phytyl chain and is therefore less hindered, thus more polar than chlorophyll *a*’ [49]. Epimers of chlorophyll and its derivatives are mostly naturally present but sometimes chlorophylls can be converted into its epimers during the extraction process. Therefore, it is anticipated that different seaweed extracts had different epimers of chlorophyll *a* derivative thus eluted at different time intervals. The most abundant product ions in ESI positive ion mass spectra of chlorophyll and its derivatives, usually relate to the dissociation of a quite weak esterifying phytyl linkage at the C-17 position of chlorophyll skeleton resulting in a fragmentation with the loss of the phytyl chain (as the phytadiene, C_20_H_38_) which appeared in the mass spectrum at the *m*/*z* value corresponding to [M + H − 278]^+^ [48,50]. The MS spectra showed the precursor ion [M + H]^+^ at *m*/*z* 893.5 and the fragment ions detected at *m*/*z* 615.2 correlating to the loss of the phytyl chain [M − 278]+ (Table 2). On the contrary, chlorophyll *b* derivative was detected only in *L. digitata* extract which was identified by absorption spectra of peak 6 (*t*_R_ 10.4 min) with the *λ*_max_ at 411 nm, 484 nm and 507 nm, and protonated molecular ion [M + H]^+^ at *m*/*z* 905.5. The presence of chlorophyll *b* derivative was confirmed by the fragment ions *m*/*z* 629.2 correlating to the removal of the phytyl chain from the chlorophyll skeleton (Table 2). Chlorophyll *b* has 14 u higher molecular weight than chlorophyll *a* because of the presence of formyl group (–CHO) instead of a methyl (–CH_3_) group at the C-7 position in chlorophyll skeleton. The presence of aldehyde group increases the polarity of chlorophyll *b* thus elutes prior to chlorophyll a on a non-polar C-18 column [49]. The identification of chlorophyll compounds was carried out as reported by Zvezdanović et al. [50] who described a similar fragmentation pattern of chlorophyll derivatives using ESI-MS/MS.

Brown seaweeds are a valuable source of lipophilic antioxidants and these compounds have a tendency to dissolve in low polarity solvents and are considered to be lipophilic in nature [43]. Our results revealed that the extraction yield in the extracts from *n*-hexane (least polar) was significantly higher in all the seaweed, however, the same extracts exhibited the lowest antioxidant capacity and phytochemical constituents. Furthermore, extracts from Mix 4 showed lower extraction yield but displayed the highest antioxidant capacity and phytochemical constituents. This indicated that polarity of an extraction solvent has no direct relation with the extraction yield and antioxidant activity, and a selective solvent system (with optimum polarity) is required to extract lipophilic antioxidant compounds from seaweed. On a contrary, Matanjun [26] reported that more polar compounds were found in seaweed extracts and increasing solvent polarity increased the extraction yield.

It was observed that *H. elongata* was better seaweed than *L. saccharina* and *L. digitata* as a source of antioxidants. Results interpret that all extracts from *H. elongata* exhibited highest antioxidant capacity (DPPH and FRAP), total phenol and flavonoid content compared to *L. saccharina* and *L. digitata*. Previous studies also reported that *Fucoid* species (*H. elongata*) contained higher phytochemical constituents and antioxidant activity than kelps (*L. saccharina* and *L. digitata*) which is in agreement with the present results [22,37,46]. The high antioxidant activities of HE may be due to the high phenolic, flavonoid and carotenoid content. The results also indicated a strong correlation between the antioxidant activity (DPPH, FRAP) and total phenolic content, which agree with study of Duan et al. [36]. On a contrary, the metal-ion chelating ability was detected higher in *L. saccharina* and *L. digitata* as compared to *H. elongata* which are in agreement with our previous findings wherein methanolic extracts from *Laminaria* species exhibited higher chelating ability than *H. elongata* [22]. Metal chelating ability in terms of ferrous ion chelating capacity is claimed as one of the important mechanisms of antioxidant activity. The ferrous ions are the most powerful pro-oxidants among various species of transition metals present in food systems [51]. Antioxidants from seaweed could either act as free radical scavengers and mitigate the ROS/free radicals [52] or could prevent the formation of hydroxyl radicals by either deactivating free metal ions through chelation or converting H_2_O_2_ to other harmless compounds (such as water and oxygen) [11].

Previous studies have reported many compounds in seaweed, for example zeaxanthin, fucoxanthin, violaxanthin, *β*-carotene, phlorotannins, anthocyanin, gallic acid, kaempferol, gallic acid 4-*O*-glucoside, cirsimaritin, carnosic acid, epigallocatechin gallate, epicatechin and fatty acids, which are strong antioxidant components [11,14,39,40,41,45,53,54,55]. In this study, the antioxidant capacity in lipophilic extract were the result of pigments and phenolic compounds. Compounds such as cyanidin-3-*O*-glucoside, *β*-carotene, violaxanthin and fucoxanthin were identified in the Mix 4 extract of *H. elongata* which could be the reason that the selected seaweed exhibited the highest antioxidant capacity. Carotenoids compounds such as violaxanthin and fucoxanthin were identified in the *L. saccharina* extract while violaxanthin and cyanidin-3-*O*-glucoside were identified in the *L. digitata* extract. The extract from *H. elongata* exhibited more antioxidant compounds than *L. saccharina* and *L. digitata*, and the antioxidant capacity in 3 species follow the following order: *H. elongata* > *L. saccharina* > *L. digitata*. It is also anticipated that chlorophyll compounds were least responsible for the antioxidant capacity as Mix 4 extracts from tested species showed moderate total chlorophyll content but exhibited the highest antioxidant capacity. Lanfer-Marquez et al. [56] reported that chlorophyll derivatives shows antioxidant capacity at very high concentration by behaving as pro-oxidants. However, they do not seem to donate hydrogen when exhibiting antioxidant capacity but may be involved in protection of linoleic acid against oxidation or by preventing breakdown of hydroperoxides. The study screened a selective solvent system for extracting lipophilic antioxidants and identified a range of antioxidant compounds. The identification of these lipophilic antioxidant compounds in selected brown seaweeds, can constitute a new move in the understanding of the health benefits of Irish brown seaweed as functional ingredients in food, cosmetics and medicinal preparation.

## 4. Conclusions

In conclusion, lipophilic extracts from Irish brown seaweed *H. elongata*, *L. saccharina* and *L. digitata* exhibit strong antioxidant property and metal-ion chelating ability. The phytochemical content and antioxidant capacity were majorly affected by the polarity or dielectric constant of extraction solvents. The highest phytochemical content and antioxidant capacity were achieved by an equal volume mixture of *n*-hexane, diethyl ether and chloroform (Mix 4) in all the seaweed studied. Among all the tested species, *H. elongata* was the most potent species which contained the highest antioxidant capacity followed by *L. saccharina* and *L. digitata*. The antioxidant capacity of *H. elongata* was comparable with that of reference ascorbic acid. A total of 10–11 lipophilic compounds with potential antioxidant capacity across the tested seaweed were identified by comparing retention times and UV spectral data. LC-ESI-MS/MS based characterization of lipophilic extracts confirmed the presence of fucoxanthin, violaxanthin, *β*-carotene, cyanidin-3-*O*-glucoside and other carotenoid and chlorophyll derivatives in the extracts. This suggests that algal derived lipophilic antioxidants may be the principal constituents responsible for the antioxidant properties from these species. These findings indicate that there may be a potential to further characterize these compounds in such extracts which can be used in pharmaceuticals, foods and cosmetics to act as antioxidants thus enhancing the quality and nutritive value of such products. Although seaweed has a great potential to be used as a source of natural antioxidant in food and cosmetics, their application as a dietary supplement or as a food ingredient should not be based only on in-vitro analysis which is just a preliminary screening tool. More research focusing on mechanisms of antioxidant action and activity against various free radicals will be advantageous in leading to the development of food and medicinal products to protect against certain age-related diseases. The identified lipophilic compounds/extracts should also be screened for their toxicity as well as for bioavailability and bioaccessibility in an in-vivo system prior to their application in commercial products.

## Figures and Tables

**Table 1:** Extraction yield and phytochemical content of lipophilic extracts of brown seaweed obtained from various organic solvents and their mixtures (semi/non-polar solvents).

Organic	Dielectric	Yield	TPC	TFC	TCC	TChC
Solvents	Constant (ε)	%	mg GAE/g	mg QE/g	(μg/g)	(µg/g)
***H. elongata***						
*n*-hexane	2.0	0.20 ± 0.02 ^a,p^	14.1 ± 0.79 ^a^	11.3 ± 2.50 ^a^	1.55 ± 0.12 ^a^	3.23 ± 1.01 ^a^
Diethyl ether	4.3	0.17 ± 0.01 ^b^	165.2 ± 1.46 ^b^	92.1 ± 5.64 ^b^	2.18 ± 0.93 ^b^	2.41 ± 1.00 ^b^
Chloroform	5.0	0.11 ± 0.01 ^c^	71.2 ± 2.33 ^c^	37.5 ± 3.75 ^c^	2.81 ± 1.03 ^c^	6.62 ± 1.34 ^c^
Mix 1	3.2	0.05 ± 0.02 ^e^	121.5 ± 3.67 ^d^	60.4 ± 4.02 ^d^	1.79 ± 0.22 ^d^	1.70 ± 0.91 ^d^
Mix 2	3.5	0.16 ± 0.03 ^b,d^	152.3 ± 1.98 ^e^	55.8 ± 2.60 ^d^	1.93 ± 0.31 ^d^	1.56 ± 0.61 ^d^
Mix 3	4.7	0.16 ± 0.02 ^b^	88.9 ± 2.96 ^f^	85.8 ± 3.82 ^b^	2.66 ± 1.04 ^e^	3.68 ± 1.12 ^e^
Mix 4 *	3.8	0.14 ± 0.01 ^d^	180.2 ± 1.84 ^g,p^	131.3 ± 4.51 ^e,p^	3.15 ± 0.91 ^f,p^	3.86 ± 1.22 ^e,p^
***L. saccharina***						
*n*-hexane	2.0	0.19 ± 0.03 ^a,q^	9.5 ± 1.93 ^a^	6.9 ± 0.88 ^a^	1.45 ± 0.42 ^a^	4.66 ± 1.03 ^a^
Diethyl ether	4.3	0.12 ± 0.01 ^b^	53.4 ± 0.96 ^b^	33.1 ± 2.65 ^b^	2.14 ± 0.83 ^b^	3.48 ± 0.93 ^b^
Chloroform	5.0	0.08 ± 0.02 ^c,d^	12.5 ± 0.32 ^c^	7.5 ± 0.00 ^a^	2.59 ± 0.91 ^c^	7.82 ± 1.54 ^c^
Mix 1	3.2	0.06 ± 0.01 ^d^	29.1 ± 0.64 ^d^	17.5 ± 1.77 ^c^	1.86 ± 0.63 ^d^	2.75 ± 1.08 ^d^
Mix 2	3.5	0.11 ± 0.04 ^b^	25.2 ± 1.61 ^e^	15.0 ± 3.54 ^c^	2.08 ± 0.39 ^b^	2.46 ± 1.17 ^e^
Mix 3	4.7	0.09 ± 0.01 ^c^	39.8 ± 1.61 ^f^	22.5 ± 1.77 ^d^	2.73 ± 0.84 ^e^	3.48 ± 1.32 ^b^
Mix 4 *	3.8	0.07 ± 0.01 ^c,d^	73.4 ± 0.32 ^g,q^	56.3 ± 1.77 ^e,q^	2.75 ± 0.88 ^e,q^	3.62 ± 1.22 ^f,p^
***L. digitata***						
*n*-hexane	2.0	0.17 ± 0.02 ^a,r^	7.7 ± 0.64 ^a^	4.4 ± 0.88 ^a^	1.20 ± 1.24 ^a^	6.19 ± 1.42 ^a^
Diethyl ether	4.3	0.14 ± 0.02 ^a,d^	48.9 ± 2.25 ^b^	29.4 ± 2.65 ^b^	1.35 ± 1.64 ^b^	5.65 ± 1.64 ^b^
Chloroform	5.0	0.09 ± 0.01 ^b,c^	15.2 ± 2.25 ^c^	8.1 ± 0.88 ^c^	1.93 ± 1.17 ^c^	8.86 ± 1.93 ^c^
Mix 1	3.2	0.08 ± 0.02 ^c^	29.1 ± 2.57 ^d^	18.1 ± 0.88 ^d^	1.39 ± 1.28 ^b^	4.62 ± 1.76 ^d^
Mix 2	3.5	0.11 ± 0.04 ^b^	29.5 ± 2.57 ^e^	18.1 ± 2.65 ^d^	1.44 ± 0.68 ^d^	2.47 ± 1.48 ^e^
Mix 3	4.7	0.12 ± 0.00 ^b,d^	46.8 ± 2.57 ^f^	26.3 ± 1.77 ^b,e^	1.43 ± 1.29 ^d^	2.71 ± 1.39 ^f^
Mix 4 *	3.8	0.11 ± 0.02 ^b^	52.7 ± 1.93 ^g,r^	31.9 ± 2.65 ^e,r^	2.19 ± 1.37 ^e,r^	2.88 ± 1.08 ^f,q^

Values are expressed as mean ± standard deviation (SD). Values within a species with different letters (a–g) in columns are significantly different (*p* < 0.05), n = 6. * Values among the three species with different letters (p–r) in columns, are significantly different (*p* < 0.05). Yield (%) is calculated in terms of g of dry extracts/100 g of fresh weight. TPC (total phenolic content) and TFC (total flavonoid content) are expressed as mg gallic acid equivalents/g (dw) and mg quercetin equivalents/g (dw), respectively. TCC (total carotenoid content) and TChC (total chlorophyll content) are reported in µg/g (dw). Mix 1: *n*-hexane and diethyl ether; Mix 2: *n*-hexane and chloroform; Mix 3: diethyl ether and chloroform; Mix 4: *n*-hexane, diethyl ether and chloroform. All the solvents were mixed in 1:1 or 1:1:1 (*v*/*v*/*v*) ratio.

**Table 2:** UV/visible (*λ*_max_) and characteristic mass spectra (MS/MS) of the compounds isolated from lipophilic extracts of brown seaweeds *H. elongata*, *L. saccharina* and *L. digitata.*

Peak No	*t*_R_ [min]	*λ*_max_ [nm]	Molecular Ion Species M^+^ [*m*/*z*]	Fragment Ions (MS–MS) [*m*/*z*]	Identification
***H. elongata***					
1	2.1	269,424	--	--	Unidentified
2	2.5	282,532	449 [M + H]^+^	287 [M + H − 162]^+^	Cyanidin-3-*O*-glucoside
3	3.2	326,425	--	--	Carotenoid
4	3.8	453,480	536.9 [M + H]^+^	444.2 [M + H − 92]^+^ 430.3 [M + H − 106]^+^	*β*-carotene
5	5.3	272,408,505	601.5 [M + H]^+^	583.5 [M + H − 18]^+^ 565.5 [M + H − 18 − 18]^+^	Violaxanthin
6	6.2	278,430,620,662	893.5 [M + H]^+^	615.2 [M + H − 278]^+^	Chlorophyll *a* derivative
7	8.5	276,420,446	--	--	Carotenoid
8	9.8	275,430,592,664	--	--	Chlorophyll
9	11.5	273,423,513		--	Carotenoid
10	13.5	327,420,472,505	--	--	Carotenoid
11	15.4	266,332,448	659.6 [M + H]^+^	641.6 [M + H − 18]^+^ 581.5 [M + H − 78]^+^	Fucoxanthin
12	24.1	278,422,508	--	--	Carotenoid
***L. saccharina***					
1	1.9	278,424	--	--	Unidentified
2	2.5	326,425	--	--	Carotenoid
3	3.0	269,425	--	--	Unidentified
4	3.4	424,572	--	--	Carotenoid
5	3.8	430,620,662	893.5 [M + H]^+^	615.2 [M + H − 278]^+^	Chlorophyll *a* derivative
6	4.2	272,425,532	--	--	Carotenoid
7	5.0	276,413,504	601 [M + H]^+^	583 [M + H − 18]^+^ 565 [M + H − 18 − 18]^+^	Violaxanthin
8	5.9	422,532	--	--	Carotenoid
9	8.3	276,424,504	--	--	Carotenoid
10	8.9	431,483,665	--	--	Chlorophyll
11	10.9	421,572			Carotenoid
12	12.6	266,332,448	659.2 [M + H]^+^	641.2 [M + H − 18]^+^ 581.5 [M + H − 78]^+^	Fucoxanthin
13	19.7	276,429,527	--	--	Carotenoid
***L. digitata***					
1	1.8	282,532	449 [M + H]^+^	287 [M + H − 162]^+^	Cyanidin-3-*O*-glucoside
2	4.4	425,572	--	--	Carotenoid
3	5.6	273,410,505	601.5 [M + H]^+^	583.5 [M + H − 18]^+^ 565.5 [M + H − 18 − 18]^+^	Violaxanthin
4	7.4	320,531	--	--	Unidentified
5	8.1	425,483,529	--	--	Carotenoid
6	9.4	451,484,507,641	906.2 [M + H]^+^	628.2 [M + H − 278]^+^	Chlorophyll *b* derivative
7	10.4	275,430,620,662	893.5 [M + H]^+^	615.2 [M + H − 278]^+^	Chlorophyll *a* derivative
8	11.1	328,536	--	--	Unidentified
9	13.0	277,359,420	--	--	Carotenoid
10	13.7	425,572	--	--	Carotenoid
11	15.9	274,376,427,527	--	--	Carotenoid
12	18.8	274,399,429,528	--	--	Carotenoid

## References

[1] Shahidi F., Naczk M. (2004). Phenolics in Food and Nutraceuticals.

[2] Prior R., Wu X., Schaich K. (2005). Standardized methods for the determination of antioxidant capacity and phenolics in foods and dietary supplements. J. Agric. Food Chem..

[3] Lee H.-H., Lin C.-T., Yang L.-L. (2007). Neuroprotection and free radical scavenging effects of Osmanthus fragrans. J. Biomed. Sci..

[4] Augustyniak A., Bartosz G., Čipak A., Duburs G., Horáková L.U., Łuczaj W., Majekova M., Odysseos A.D., Rackova L., Skrzydlewska E. (2010). Natural and synthetic antioxidants: An updated overview. Free Radic. Res..

[5] Shahidi F. (2009). Nutraceuticals and functional foods: Whole versus processed foods. Trends Food Sci. Technol..

[6] Rice-Evans C.A., Miller N.J., Paganga G. (1996). Structure-antioxidant activity relationships of flavonoids and phenolic acids. Free Radic. Biol. Med..

[7] Lesser M.P. (2006). Oxidative stress in marine environments: Biochemistry and physiological ecology. Annu. Rev. Physiol..

[8] Plaza M., Santoyo S., Jaime L., Garcia-Blairsy Reina G., Herrero M., Senorans F.J., Ibanez E. (2010). Screening for bioactive compounds from algae. J. Pharm. Biomed. Anal..

[9] Ganesan P., Kumar C.S., Bhaskar N. (2008). Antioxidant properties of methanol extract and its solvent fractions obtained from selected Indian red seaweeds. Bioresour. Technol..

[10] Herrero M., Jaime L., Martín-Álvarez P.J., Cifuentes A., Ibáñez E. (2006). Optimization of the Extraction of Antioxidants from Dunaliella salina Microalga by Pressurized Liquids. J. Agric. Food Chem..

[11] Huang H.-L., Wang B.-G. (2004). Antioxidant Capacity and Lipophilic Content of Seaweeds Collected from the Qingdao Coastline. J. Agric. Food Chem..

[12] Maeda H., Tsukui T., Sashima T., Hosokawa M., Miyashita K. (2008). Seaweed carotenoid, fucoxanthin, as a multi-functional nutrient. Asia Pac. J. Clin. Nutr..

[13] Balboa E.M., Conde E., Moure A., Falqué E., Domínguez H. (2013). In vitro antioxidant properties of crude extracts and compounds from brown algae. Food Chem..

[14] Rajauria G., Foley B., Abu-Ghannam N. (2017). Characterization of dietary fucoxanthin from Himanthalia elongata brown seaweed. Food Res. Int..

[15] Focsan A.L., Polyakov N.E., Kispert L.D. (2017). Photo protection of Haematococcus pluvialis algae by astaxanthin: Unique properties of astaxanthin deduced by EPR, optical and electrochemical studies. Antioxidants.

[16] Zaragozá M.C., López D., Sáiz M.P., Poquet M., Pérez J., Puig-Parellada P., Marmol F., Simonetti P., Gardana C., Lerat Y. (2008). Toxicity and antioxidant activity in vitro and in vivo of two Fucus vesiculosus extracts. J. Agric. Food Chem..

[17] Naczk M., Shahidi F. (2004). Extraction and analysis of phenolics in food. J. Chromatogr. A.

[18] Çelik S.E., Özyürek M., Güçlü K., Apak R. (2010). Solvent effects on the antioxidant capacity of lipophilic and hydrophilic antioxidants measured by CUPRAC, ABTS/persulphate and FRAP methods. Talanta.

[19] Rajauria G., Jaiswal A.K., Abu-Ghannam N., Gupta S. (2013). Antimicrobial, antioxidant and free radical-scavenging capacity of brown seaweed Himanthalia elongata from western coast of Ireland. J. Food Biochem..

[20] Rajauria G., Abu-Ghannam N. (2013). Isolation and partial characterization of bioactive fucoxanthin from Himanthalia elongata brown seaweed: A TLC-based approach. Int. J. Anal. Chem..

[21] Liu S., Lin J., Wang C., Chen H., Yang D. (2009). Antioxidant properties of various solvent extracts from lychee (Litchi chinenesis Sonn.) flowers. Food Chem..

[22] Rajauria G., Jaiswal A.K., Abu-Ghannam N., Gupta S. (2010). Effect of hydrothermal processing on colour, antioxidant and free radical scavenging capacities of edible Irish brown seaweeds. Int. J. Food Sci. Technol..

[23] Jaiswal A.K., Rajauria G., Abu-Ghannam N., Gupta S. (2012). Effect of Different Solvents on Polyphenolic Content, Antioxidant Capacity and Antibacterial Activity of Irish York Cabbage. J. Food Biochem..

[24] Decker E.A., Welch B. (1990). Role of ferritin as a lipid oxidation catalyst in muscle food. J. Agric. Food Chem..

[25] Sugawara T., Baskaran V., Tsuzuki W., Nagao A. (2002). Brown algae fucoxanthin is hydrolyzed to fucoxanthinol during absorption by Caco-2 human intestinal cells and mice. J. Nutr..

[26] Matanjun P., Mohamed S., Mustapha N.M., Muhammad K., Ming C.H. (2008). Antioxidant activities and phenolics content of eight species of seaweeds from north Borneo. J. Appl. Phycol..

[27] Pangestuti R., Kim S.-K. (2011). Biological activities and health benefit effects of natural pigments derived from marine algae. J. Funct. Foods.

[28] Peng J., Yuan J.-P., Wu C.-F., Wang J.-H. (2011). Fucoxanthin, a marine carotenoid present in brown seaweeds and diatoms: Metabolism and bioactivities relevant to human health. Mar. Drugs.

[29] Chandini S.K., Ganesan P., Bhaskar N. (2008). In vitro antioxidant activities of three selected brown seaweeds of India. Food Chem..

[30] Hosokawa M., Okada T., Mikami N., Konishi I., Miyashita K. (2009). Bio-functions of marine carotenoids. Food Sci. Biotechnol..

[31] Mhadhebi L., Mhadhebi A., Robert J., Bouraoui A. (2014). Antioxidant, anti-inflammatory and antiproliferative effects of aqueous extracts of three mediterranean brown seaweeds of the genus cystoseira. Iran. J. Pharm. Res. IJPR.

[32] Gammone M.A., D’Orazio N. (2015). Anti-obesity activity of the marine carotenoid fucoxanthin. Mar. Drugs.

[33] Abdul F., Singh I.M.P. (2009). Effect of ternary solvent system on the permeability of lisinopril across rat skin in vitro. Int. J. Drug Dev. Res..

[34] Sahreen S., Khan M.R., Khan R.A. (2010). Evaluation of antioxidant activities of various solvent extracts of Carissa opaca fruits. Food Chem..

[35] Sun T., Ho C.T. (2005). Antioxidant activities of buckwheat extracts. Food Chem..

[36] Duan X.-J., Zhang W.-W., Li X.-M., Wang B.-G. (2006). Evaluation of antioxidant property of extract and fractions obtained from a red alga, Polysiphonia urceolata. Food Chem..

[37] Jiménez-Escrig A., Jiménez-Jiménez I., Pulido R., Saura-Calixto F. (2001). Antioxidant activity of fresh and processed edible seaweeds. J. Sci. Food Agric..

[38] Gordon M.H., Kourimská L. (1995). Effect of antioxidants on losses of tocopherols during deep-fat frying. Food Chem..

[39] Maoka T., Fujiwara Y., Hashimoto K., Akimoto N. (2002). Rapid Identification of Carotenoids in a Combination of Liquid Chromatography/UV-Visible Absorption Spectrometry by Photodiode-Array Detector and Atomospheric Pressure Chemical Ionization Mass Spectrometry (LC/PAD/APCI-MS). J. Oleo Sci..

[40] Schütz K., Persike M., Carle R., Schieber A. (2006). Characterization and quantification of anthocyanins in selected artichoke (Cynara scolymus L.) cultivars by HPLC–DAD–ESI–MS n. Anal. Bioanal. Chem..

[41] Heriyanto J.A., Shioi Y., Limantara L., Brotosudarmo T. (2017). Analysis of pigment composition of brown seaweeds collected from Panjang Island, Central Java, Indonesia. Philipp. J. Sci..

[42] Meléndez-Martínez A.J., Britton G., Vicario I.M., Heredia F.J. (2007). Relationship between the colour and the chemical structure of carotenoid pigments. Food Chem..

[43] Schoefs B. (2002). Chlorophyll and carotenoid analysis in food products. Properties of the pigments and methods of analysis. Trends Food Sci. Technol..

[44] Gouvêa A.C., Araujo M.C., Schulz D.F., Pacheco S., Godoy R.L., Cabral L.M. (2012). Anthocyanins standards (cyanidin-3-*O*-glucoside and cyanidin-3-*O*-rutinoside) isolation from freeze-dried açaí (*Euterpe oleraceae* Mart.) by HPLC. Food Sci. Technol..

[45] Rivera S.M., Christou P., Canela-Garayoa R. (2014). Identification of carotenoids using mass spectrometry. Mass Spectrom. Rev..

[46] De Quiros A.R.-B., Frecha-Ferreiro S., Vidal-Pérez A.M., López-Hernández J. (2010). Antioxidant compounds in edible brown seaweeds. Eur. Food Res. Technol..

[47] Enzell C., Francis G., Liaaen-Jensen S. (1968). Mass spectrometric studies of carotenoids. I. Occurrence and intensity ratios of M–92 and M–106 peaks. Acta Chem. Scand..

[48] Milenković S.M., Zvezdanović J.B., Anđelković T.D., Marković D.Z. (2012). The identification of chlorophyll and its derivatives in the pigment mixtures: HPLC-chromatography, visible and mass spectroscopy studies. Adv. Technol..

[49] Lim C.K. (2009). High-Performance Liquid Chromatography and Mass Spectrometry of Porphyrins, Chlorophylls and Bilins.

[50] Zvezdanović J.B., Petrović S.M., Marković D.Z., Andjelković T.D., Andjelković D.H. (2014). Electrospray ionization mass spectrometry combined with ultra high performance liquid chromatography in the analysis of in vitro formation of chlorophyll complexes with copper and zinc. J. Serb. Chem. Soc..

[51] Hultin H. (1994). Oxidation of lipids in seafoods. Seafoods: Chemistry, Processing Technology and Quality.

[52] Molyneux P. (2004). The use of the stable free radical diphenylpicrylhydrazyl (DPPH) for estimating antioxidant activity. Songklanakarin J. Sci. Technol..

[53] Santoso J., Yoshie Y., Suzuki T. (2004). Polyphenolic compounds from seaweeds: Distribution and their antioxidative effect. Dev. Food Sci..

[54] Takeshi S., Yumiko Y.-S., Joko S. (2005). Mineral components and anti-oxidant activities of tropical seaweeds. J. Ocean Univ. China.

[55] Rajauria G., Foley B., Abu-Ghannam N. (2016). Identification and characterization of phenolic antioxidant compounds from brown Irish seaweed Himanthalia elongata using LC-DAD–ESI-MS/MS. Innov. Food Sci. Emerg. Technol..

[56] Lanfer-Marquez U.M., Barros R.M., Sinnecker P. (2005). Antioxidant activity of chlorophylls and their derivatives. Food Res. Int..

